# La conjonctive tarsale une localisation exceptionnelle du carcinome épidermoïde conjonctival

**DOI:** 10.11604/pamj.2018.30.139.11216

**Published:** 2018-06-19

**Authors:** Bennis Ahmed, Benatiya Andaloussi Idriss

**Affiliations:** 1 Faculté de Médecine et de Pharmacie de Fès, CHU Hassan II, Fès, Maroc

**Keywords:** Conjonctive, carcinome épiderpoïde, exérèse tumorale, Conjunctiva, squamous carcinoma, tumor resection

## Abstract

We here report the case of a 48-year old patient with a family history of five deaths due to cancers with different localizations, presenting with ulcero-budding nodular swelling of the lower palpebral conjunctiva measuring 2cm long its longer axis, gradually increasing volume over one year. It involved the inner half of the lower tarsal conjunctiva closely with the lower lacrimal punctum (A). Tumor biopsy showed well differentiated squamous carcinoma of the conjunctiva. Treatment was based on tumor resection (B) with wide negative safety margins on anatomopathological exam.

## Image en médecine

Patient de 48 ans de teint clair, ayant la notion de 5 décès dans la famille par des cancers de localisations variables, qui présente depuis une année une tuméfaction de la conjonctive palpébrale inférieur augmentant progressivement de volume, de forme nodulaire ulcéro-bourgennante, mesurant 2cm de grand axe, occupant la moitié interne de la conjonctive tarsale inférieure présentant un contact intime avec le point lacrymal inférieur (A). La biopsie de la tumeur est en faveur d’un carcinome épidermoïde de la conjonctive bien différencié. Le traitement a consisté en une exérèse tumorale (B) avec marges de sécurité larges revenant saines après étude anatomopathologique.

**Figure 1:**
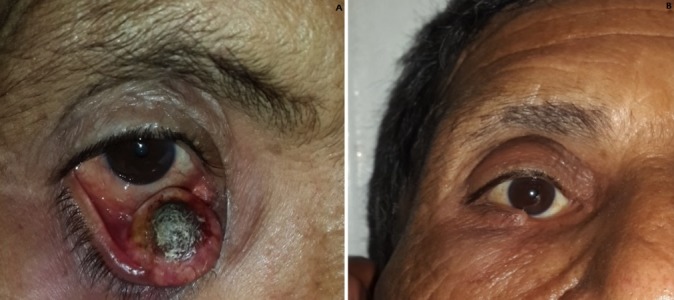
A) tuméfaction de la conjonctive palpébrale inférieur augmentant progressivement de volume, de forme nodulaire ulcéro-bourgennante, mesurant 2cm de grand axe, occupant la moitié interne de la conjonctive tarsale inférieure présentant un contact intime avec le point lacrymal inférieur avant chirurgie; B) aspect après exérèse tumorale avec respect des marges de sécurité après chirurgie

